# The epidemiologic case for urban health: conceptualizing and measuring the magnitude of challenges and potential benefits

**DOI:** 10.12688/f1000research.154967.1

**Published:** 2024-08-22

**Authors:** Michael D. Garber, Tarik Benmarhnia, Audrey de Nazelle, Mark Nieuwenhuijsen, David Rojas-Rueda

**Affiliations:** 1 Scripps Institution of Oceanography, University of California San Diego, San Diego, California, USA; 2 Irset Institut de Recherche en Santé, Environnement et Travail, University of Rennes, Rennes, France; 3 Imperial College London Centre for Environmental Policy, London, England, UK; 4 MRC Centre for Environment and Health, Imperial College London School of Public Health, London, England, UK; 5 Barcelona Institute for Global Health (ISGlobal), Barcelona, Spain; 6 Universitat Pompeu Fabra, Barcelona, Catalonia, Spain; 7 CIBER Epidemiología y Salud Pública (CIBERESP), Madrid, Spain; 8 Department of Environmental & Radiological Health Sciences, Colorado State University, Fort Collins, Colorado, USA; 9 Colorado State University, Colorado School of Public Health, Colorado, USA

**Keywords:** Urban health, Epidemiology, Global health, Policy evaluation, Public health surveillance

## Abstract

We discuss how epidemiology has been and can continue to be used to advance understanding of the links between urban areas and health informed by an existing urban-health conceptual framework. This framework considers urban areas as contexts for health, determinants of health and modifiers of health pathways, and part of a complex system that affects health. We highlight opportunities for descriptive epidemiology to inform the context of urban health, for example, by characterizing the social and physical environments that give rise to health and the actions that change those conditions. We then describe inferential tools for evaluating the impact of group-level actions (e.g., interventions, policies) on urban health, providing some examples, and describing assumptions and challenges. Finally, we discuss opportunities and challenges of applying systems thinking and methods to advance urban health. While different conceptual frames lead to different insights, each perspective demonstrates that urban health is a major and growing challenge. The effectiveness of urban health knowledge, action, and policy as the world continues to urbanize can be informed by applying and expanding upon research and surveillance methods described here.

## Key messages

• The health of urban residents is a major component of global health and will become more important with continuing urbanization.
• Urban areas share common health-relevant environmental and social features, but the health of urban residents and the impact of urban living on health vary between and within cities.
• As a previously published conceptual framework articulates, the links between urban environments and population health can be conceptualized in various ways, including as the setting in which population health occurs, as a cause of population health, and as part of a complex system that shapes population health. Each conceptualization raises distinct challenges and opportunities regarding measurement of scale and impacts.
• Classical tools of descriptive epidemiology remain important for the study of urban health, and more complete health-outcome data on health is needed at the city- and within-city scales.
• Where direct observation is not feasible, health-impact modeling and small-area estimation show promise for advancing the description of urban health outcomes at high spatial resolution.
• There is a need for higher resolution and more systematic measurement of the physical, social, and policy environments that affect urban health, a task that can be considered within the purview of descriptive epidemiology.
• Research on the impact of actions and interventions on urban health and health equity interventions is limited. Evaluating these actions using longitudinal methods for group-level data should continue.

## 1. Introduction

### 1.1 The importance of urban health

As the proportion of humanity living in cities continues to rise, the health of people living in urban areas will represent an increasingly important component of global health. ^1^ In 2018, the share of the world’s population residing in urban areas was 55% and is expected to increase to 60% by 2030. ^1^ Urbanization (the process of an area becoming more urban) is accelerating in particular in low- and middle-income countries in Asia and Africa. ^1^ A clear implication of global urbanization trends is that improving public health globally will require a major focus on health in urban areas. ^2^ However, the scale of current and future urban health challenges and opportunities is not well characterized and depends on assumptions about the nature of the relationships between cities and health and the scope of urban health action.

Urban areas present both challenges and opportunities for public health. ^3^^–^^5^ Compared with rural areas, urban areas can promote health through higher standards of living and better access to resources and services, including healthcare services. ^6^^–^^8^ Nevertheless, characteristics common to many urban environments can also harm health, including air pollution; ^9^ noise pollution; heat exacerbated by urban heat islands; ^10^ inadequate green space; high availability of nutrient-poor, calorie-dense food; and unsafe transportation systems resulting in road traffic injuries and discouraging physical activity. ^6^^,^^11^ Urban areas are also vulnerable to some infectious diseases. Some, such as COVID-19, ^12^ may spread rapidly in areas with high population density and connectivity. ^11^ Others, such as malaria, have historically been more common in rural areas, ^13^ but climate change and landscape modifications including deforestation are changing spatial patterns of vector-borne diseases, increasing their occurrence in urban areas. ^14^

While urban areas can share common health-relevant environmental and social features, cities are distinct, and the health of urban residents—and the impact of urban living on health—varies both between and within cities. ^15^^,^^16^ Variable exposure to environmental conditions (both social and physical) gives rise to health inequities in urban populations by characteristics such as income and wealth, residential neighborhood, race and ethnicity, age, disability, gender identity, sexual orientation, or migration status. ^16^ Ultimately, variation in health between and within cities reveals an opportunity: urban areas can be designed and managed to promote health and health equity. Furthermore, in the context of global climate change, strategies to improve health in cities can have co-benefits for climate-change mitigation and adaptation. ^17^^–^^19^ Given this opportunity, there is a need for a clearer understanding of how urban environments and the forces that change them affect public health and of the scale of the health challenges and opportunities inherent in the urban context.

### 1.2 The role of epidemiology

Many disciplines will contribute to this needed understanding, and epidemiologists, those who study the distribution and determinants of health in populations, are well-equipped to contribute in several ways. Some tools from epidemiology, in particular descriptive epidemiology, have an established history of informing urban health. Other more contemporary topics in epidemiology, such as methods for causal inference, have yet to realize their potential in helping to answer urban-health questions. At the same time, the discipline may be well-suited to advance urban-health knowledge in ways traditionally not under its purview, such as the surveillance of the urban physical and policy environment. In this article, we discuss how epidemiology has been and can continue to be used to advance understanding of the links between urban areas and health informed by an existing urban-health conceptual framework. We then propose actions needed to continue to advance this understanding.

## 2. Conceptualizing and measuring urban health

### 2.1 A framework relating urban areas and health

Urban-health scholars have proposed various frameworks to represent and explain the links between urban environments and population health. ^6^^,^^20^^–^^25^ In the framework by Diez Roux presented in Chapter 3 of the *Urban Public Health* text, the links are conceptualized in four inter-related ways. ^24^ First, urban areas are contexts for health. That is, they are settings in which population health occurs. Second, urban areas are causes or determinants of health. These place-based causes occur at various levels, for example, the neighborhood (e.g., features of its built environment) or the city (e.g., housing policy). Third, and related to the second, urban areas can modify or reinforce individual-level health pathways. For example, genetic susceptibility to obesity can be modified by the neighborhood food environment. ^26^ Fourth—and encompassing each of the above—urban areas are part of complex systems that shape population health. ^6^^,^^21^^,^^27^ Measuring the magnitude of urban-health challenges through each of these lenses has value for researchers, policymakers, and advocates.

### 2.2 Urban areas as contexts for health

*2.2.1 Describing health-related states and behaviors in urban populations*

The most conceptually straightforward link between urban areas and health is the occurrence of diseases, health-related states, and behaviors among individuals living in urban areas. Measuring these outcomes in urban areas is important for planning and resource allocation, monitoring progress on health targets over time, comparing the distribution of health between groups, generating hypotheses, and identifying opportunities for intervention. This task has traditionally been undertaken by public health surveillance systems (such as vital statistics registries and large-scale cross-sectional surveys), cohort studies, and other tools of descriptive epidemiology. For example, national registries have been used to compare cancer incidence between urban and rural areas over time in the United States. ^28^^,^^29^ Vital registration data can be standardized and harmonized to allow for cross-city comparisons, such as in the SALURBAL study that described within-country heterogeneity in life expectancy across Latin American cities. ^15^

A barrier to descriptive epidemiology in urban areas is that information is commonly collected to be representative of an administrative unit above the city (for example, of a state, province, or country). For example, the Global Burden of Disease project, which comprehensively describes the occurrence, mortality attributable to, and disability attributable to hundreds of diseases, injuries, and risk factors, presents information at the country level. ^30^ Such comprehensive descriptive epidemiology does not exist for urban areas. Assuming 55% of the world’s population lives in urban areas ^1^ and that there were 55.4 million deaths in 2019, ^31^ then a crude calculation assuming the number of deaths in urban areas is proportional to their share of the population suggests about 30.5 million deaths (0.55*55.4 million) occurred in urban areas in 2019. Currently, only crude calculations such as this are available, and global estimates have not accounted for urban-versus-rural differences in mortality for reasons such as urban-rural differences in age structure or exposure to risks. The lack of urban-specific estimates of the health burden or of the scale of the health benefits that might result from relevant interventions poses challenges for allocating appropriate financial, institutional, and human resources to urban health action.

While more work is needed to comprehensively describe the urban burden of ill health, existing information can serve as a useful guide. When direct measurement of health outcomes (e.g., through surveys) in cities is not feasible, two approaches hold promise for advancing descriptive epidemiology within and across urban areas: health-impact modeling and small-area estimation. Health-impact modeling is often used to project the health impacts of alternative scenarios. It can also be used to estimate the burden of dimensions of health attributable to the status-quo scenario. For example, health-impact modeling was used to estimate that exposure to air pollution among residents of 13,160 urban areas globally resulted in 1.8 million excess deaths in 2019. ^9^ While valuable, health-impact modeling studies can rely on strong assumptions, for example that health effects estimated in one location can apply to the area of interest. As we discuss further below, health effects can be context-dependent. ^24^ The validity of health-impact assessment may benefit from more explicitly considering the extent to which effects are transportable from one context to another. ^32^

As health outcomes can vary considerably between and within urban areas, ^16^ methods that aim to estimate health outcomes at a fine geographic scale are vitally important. Small-area estimation (SAE) is a suite of such methods. SAE methods estimate outcome measures (e.g., disease prevalence) at a unit of geography beneath the level where the measure is known (e.g., from a U.S. state to a county). ^33^ SAE methods include, for example, non-parametric methods that use measured associations between the outcome and demographic characteristics at the larger geographic unit (e.g., U.S. states) and apply those associations to smaller units where the outcome is not measured (such as counties), spatial data smoothing, and regression techniques using area-specific data as predictors. ^33^ Long used to estimate the prevalence of health-related behaviors at the U.S. county level, ^34^^–^^36^ SAE methods have recently been used to estimate life expectancy of residents of U.S. census tracts (a within-city unit). ^37^ Results suggest there is considerable variation in life expectancy between census tracts, underscoring that health varies within cities. ^38^ An advantage of SAE methods is that they borrow information from the higher level, allowing investigations of small areas (such as an urban neighborhood) to use fewer resources than they might require if the higher-level information were not available. As with most any method, estimation relies on assumptions, for example, that the association between demographics and the outcome at the larger area holds at the smaller area. In the urban-health context, SAE methods may face particular challenges for key populations such as migrants or those who live in slums, groups whose health is difficult to measure. ^39^^,^^40^ A small-area estimate aiming to estimate the health distribution of refugee settlements may be uninformative, for example, if it uses as key inputs the health distribution of surrounding areas, which may not be a strong predictor of the health of a group of individuals who recently arrived to that area.

*2.2.2 Describing the health-relevant context of urban areas*

Descriptive epidemiology conventionally measures health outcomes (such as cancer incidence and death) and individual-level risk factors (such as smoking and diet). As knowledge about the environmental impacts on health continues to grow, an increasingly important task for descriptive epidemiology, together with other disciplines, will be to better characterize the multi-level social and physical environments in urban areas that give rise to health. ^23^^,^^41^ As an example addressing this need, a research coalition developed a dataset covering 371 cities in Latin America with various consistently defined indicators of the social environment across economic, social, housing, governmental, institutional, and organizational domains. ^41^ They noted several challenges in developing this dataset including variation in data availability, quality, and spatial and temporal resolution and extent.

The surveillance of physical environmental conditions relevant for health has advanced considerably as data have become available from remote sensing (i.e., satellite imagery), ^42^ street-level imagery (such as Google Street View), ^43^ and open-source mapping software (such as OpenStreetMap). ^44^ For example, remote sensing has been used to measure urban green space, ^45^ street-view imagery to assess walkability and disorder of neighborhoods, ^43^^,^^46^ and OpenStreetMap to assess building quality and attributes of the roadway network. ^44^^,^^47^^,^^48^ One large-scale effort using some of these tools (OpenStreetMap, notably) is the creation of a multi-city dataset of livability indicators across several Australian cities. ^49^^,^^50^ This dataset of Australian cities is impressive for its rich information about the physical urban environment at high (within-city) spatial resolution and the comparability of its measures between cities, as constituent cities are each measured using the same methods.

As illustrated by the creation of such large-scale multi-city datasets of the built environment, modern sources of street-level imagery and mapping software have eased the objective and harmonious assessment of the physical environment at scale. Challenges remain with their completeness, accuracy, ^48^ and ongoing availability and legal use in urban health-related research. ^51^

Related to the need for a consistent dataset of indicators of the social and built environment across and within cities is the need to systematically inventory policies, interventions, projects, and other actions that could affect these indicators. As Diez Roux writes, “Cities are acting,” ^2^ but, generally speaking, the public health community lacks a robust system for collecting data on these actions. Some social health-relevant social policies, particularly those codified by law, have established databases tracking them. ^52^ For example, databases on various social policies relevant for health have been documented, covering topics such as poverty and social welfare, firearms, alcohol, and tobacco. ^52^^,^^53^ Other examples of health-relevant policy surveillance in the U.S. include monitoring of policies supportive of physical activity in the United States ^54^ and of policies related to COVID-19. ^55^ At the global level, the C40 Cities, a network of cities focused on climate-change action, have documented actions cities are implementing to adapt to climate change. ^56^

Work remains, however, in tracking the various actions—from formal legislation and regulation to local interventions—city governments and other actors are taking that could affect public health. ^57^ Interventions and actions that occur beneath the city level can be particularly difficult to track systematically. Examples include the conversion of a vacant lot to housing, the construction of a neighborhood greenway, or the closing of a coal power plant. Epidemiologists, with expertise in counting and measurement across various scales of time and place, are well-suited to contribute to the surveillance of policies, interventions, and other population-level actions that could affect public health. Crowdsourcing this information may help. ^58^ Critical data include where and when the action occurred, the geographic scale of the action, and, as applicable, the level of governance responsible. ^59^ For evaluation purposes, documenting when the intervention occurred is especially important so that methods considering intervention timing (described in the next section) can be applied.

### 2.3 Urban determinants and modifiers of health

As well as a context in which population health manifests, cities and their characteristics are determinants and modifiers of health. ^3^^,^^21^^,^^60^ The magnitude of ill health arising from urban determinants—or the potential health benefits from urban interventions—can be used to inform action that decision makers can take. Understanding how impacts vary by place, time, or other contextual factors can inform whether and how to tailor action to specific urban contexts. While there is a long tradition of theoretical and empirical research studying determinants of health at levels above the individual (such as the neighborhood, city, or institution), ^61^^–^^63^ research is less robust on the effects of specific actions and policies taken by governments and other actors on health and health equity. Methods to study the longitudinal effects of group-level interventions, such as difference-in-differences, interrupted time series, and synthetic control methods, ^64^^,^^65^ are well-suited for this purpose and have been used in recent years to study effects of policies and other actions between and within cities. When possible, these methods can take advantage of natural or quasi experiments. ^66^ Some examples of their use in the urban-health context include an assessment of the effects of keeping indoor restaurant dining closed on COVID-19 rates in large U.S. cities, ^67^ of a street-design intervention on pedestrian crashes in Mexico City, ^68^ of bicycling infrastructure on bicycling levels in Atlanta, United States, ^69^ and of a heat action plan on heat-related mortality in Montreal, Canada. ^70^ Some of these tools allow for the estimation of place-specific effects which can inform the extent to which intervention effects are modified by context. ^71^

While these methods hold promise, they also bring about challenges for use in the urban-health context. One is that urban policies often co-occur, ^52^^,^^72^ posing analytical challenges for isolating the impact of one specific action from another. Second, sometimes the area of interest has no suitable comparator over relevant characteristics, even when the comparator is a weighted average of several areas. ^73^ This issue, sometimes called “non-positivity”, could arise in particular in unequal or segregated cities. A third is difficulty characterizing where to “draw the line” particularly for actions or events whose effects may be diffuse, such as wildfires or built-environment interventions. ^69^^,^^74^^,^^75^ While these challenges warrant further attention, this family of methods is sufficiently well-developed to be useful in a range of urban settings for the evaluation of policies, actions, and climate-related events. ^74^

### 2.4 Urban health as part of a complex systems

Above, we noted that a challenge with some policy-evaluation methods is isolating specific effects in the context of other co-occurring interventions and actions. ^52^ Perhaps more importantly, isolating specific effects may not always be the most useful question to answer if the goal is to improve population health. ^76^ These conceptual and methodological challenges motivate calls to view urban interventions and actions as part of complex systems. ^27^^,^^76^^–^^78^ Features of complex systems include multiple levels of organization, heterogenous units, dependencies between units, and feedback loops. ^24^ In a review of public health interventions adopting a complex-systems perspective, the authors categorized the goals of such studies into five stages: theorizing, prediction, process evaluation, impact evaluation, and further prediction. ^79^ Information derived from considering urban health as a complex system adds a new layer of value for policy-making beyond the burden of urban ill health and the health impacts of specific urban determinants and actions. Well-executed, a complex-systems approach can help policymakers understand tradeoffs, unanticipated effects, implementation challenges, the potential impact of multiple interventions, and other effects arising from urban complexity.

Prediction (or simulation) is a common application of these complex-systems methods, the purpose of which is to “hypothesize and simulate how an intervention may impact on and interact with a complex system” or, similarly, to “hypothesize and simulate how agents within a complex system react and interact in response to an intervention.” ^79^ Respectively, these two objectives roughly correspond to the approach of system dynamic models and agent-based models (ABMs). ^79^ The strengths of these systems methods are that they allow the investigator to perform complex simulations of how an intervention may unfold, considering interactions, interdependencies, and feedback.

ABMs, in particular, have been explored for their potential to inform and evaluate interventions related to urban health. ^80^ Authors exploring their utility and challenges have suggested that ABMs are attractive for three reasons. First, the method “… is well-suited for modeling exposure influenced by specific behaviors, their timing, and location.” ^80^ Second, “any mathematical relationship— … non-linear, complex interactions—can be algorithmically modeled in an ABM.” ^80^ Third, “ABMs can integrate many factors resulting in a holistic and context-dependent model.” ^80^ ABMs also raise several challenges, however. ^80^^,^^81^ One is that they require a rich evidence base to inform their models. Evidence does not always exist to inform the various steps of such models, requiring researchers to rely on strong assumptions, and erroneous assumptions may affect model behavior in complex ways. Existing epidemiological evidence may also have been conducted in populations whose distribution of key characteristics differs from that in which the model is to be applied. These challenges imply that it remains important to conduct traditional epidemiologic research to inform these systems models.

## 3. Conclusions

### 3.1 Summary of needed understanding

The reviewed conceptual model considers various ways to understand—and even define—urban health. Within each conceptual frame, more work is needed to advance understanding of urban health and support action to improve the health of urban populations. For example, it is unknown how much ill health occurs in urban populations. In this article, we crudely estimated that about 30-35 million deaths occur annually in urban areas, but this estimate relies on strong assumptions and ought to be improved upon. More descriptive epidemiology is needed to characterize urban health with both fine (within-city) resolution and at a large (global) scale to better understand differences in health between and within urban areas. There is a need for improved measurements of both the health of urban populations and of the environments that give rise to health. Creating databases at the multi-city scale containing high-resolution indicators of the social and built environments and related policies, interventions, and other actions should be encouraged. Noted examples characterizing indicators and policies in Latin America ^41^ and Australia ^49^ may serve as a guide.

There is also a need to understand what works to improve urban health. Decades of research have shown links between social and physical urban environments and health, but research evaluating the impacts of actual actions (e.g., policies and interventions) that may affect urban health and health equity remains comparatively scarce. Monitoring actions between and within cities would enable such evaluations. Finally, the argument for considering the health effects of urban living as part of a complex system is persuasive, ^76^ but our tools for quantitatively understanding these system-level processes remain under-developed in the public-health literature, in particular for the evaluation of urban-health interventions. ^79^ That many of these systems modeling methods require information-rich empirical evidence also underscores the need for traditional epidemiologic research to inform their components. While it will take a multi-disciplinary effort to address these gaps in understanding, the discipline of epidemiology, specifically, can inform urban-health understanding by adopting a broad view of descriptive epidemiology that includes surveillance of the urban physical and policy environment, continuing to evaluate group-level interventions between and within urban areas, and advancing research on systems thinking in urban health issues.

### 3.2 Actions needed to support gaps in understanding

To support this needed understanding, we recommend several actions that can be taken by stakeholders in national governments, local governments, not-for-profit organizations, coalitions, and academia. National governments could increase funding for fine-scale urban health surveys and surveillance systems and develop dashboards displaying resulting measures. One example in the U.S. context is the City Health Dashboard. ^82^^,^^83^ As individual countries improve urban-health surveillance, the magnitude of urban-health challenges at the global or regional scale may become more apparent. Public health practitioners and other officials in local governments could similarly collect small-area health data and, ideally, present it in standardized formats on open-data portals. ^84^ Local governments are especially well-suited to conduct policy surveillance on actions that could affect urban health, as they are often responsible for their on-the-ground implementation. If data collection on all relevant interventions and actions is not feasible for government officials, they could partner with academic researchers, not-for-profit organizations, or global coalitions such as C40. ^85^ The evaluation of policies and interventions that could affect urban health at the local level is another area well-served by cross-sectoral collaboration. This collaborative evaluation research is particularly important in sectors not traditionally viewed under the public-health umbrella ranging from but not limited to transportation systems, housing policy, taxation policy, and energy systems. Throughout urban health surveillance and evaluation, care should be taken to collect data to represent marginalized groups and evaluate impacts on equity-related outcomes.

## Data Availability

No data are associated with this article.
