# Supplementary material for: The epidemiologic case for urban health: conceptualizing and measuring the magnitude of challenges and potential benefits
Source: F1000Res. 2025 Mar 17;13:950. Originally published 2024 Aug 22. [Version 2] doi: 10.12688/f1000research.154967.2 (PMC11920689; doi:10.12688/f1000research.154967.2)
Supplement: Peer review history

# Reviewer report: Reviewer response for version 2

**DOI:** 10.5256/f1000research.178856.r371416

**Published:** 2025-03-18

**Authors:** Alina Schnake-Mahl

**Affiliations:** 1 Urban Health Collaborative, Department of Health Management and Policy, Drexel Dornsife School of Public Health, Philadelphia, USA

The authors have addressed all comments. I appreciate their thorough responses.

Is the review written in accessible language?

Yes

Are all factual statements correct and adequately supported by citations?

Yes

Are the conclusions drawn appropriate in the context of the current research literature?

Yes

Is the topic of the review discussed comprehensively in the context of the current literature?

Yes

Reviewer Expertise:

social epidemiology, policy evaluation, urban health, health disparities

I confirm that I have read this submission and believe that I have an appropriate level of expertise to confirm that it is of an acceptable scientific standard.

# Reviewer report: Reviewer response for version 1

**DOI:** 10.5256/f1000research.170075.r337107

**Published:** 2024-12-04

**Authors:** Simon Rüegg

**Affiliations:** 1 University of Zurich, Zurich, Switzerland

Given that in the 21 ^st^ century most of the global population is living in urban settings, the reflection on the importance and requirements of urban epidemiology are pertinent and timely. Particularly the geographic resolution is a hindrance in open sight which must be brought to the attention of policy makers and health agents. However, given the current polycrisis including climate change, biodiversity loss and pollution which are directly and indirectly affecting human health, the sole emphasis on human health indicators seems somewhat anachronistic and narrowly focused. The authors legitimately build on historic practices in epidemiology using the four framings of cities as contexts, as determinants, as causes of human health and as complex systems that shape health.

 I would find it interesting to differentiate somewhat more explicitly, which framing is useful for which kind of questions. Admittedly I am biased by a complexity approach to health. But I would advocate such an approach primarily for wicked health problems for which multiple aspects interact. Also, in the context of sustainable development participatory and complexity-aware approaches have shown to be more effective than linear analysis and intervention. There may however be public health related questions, e.g. health care infrastructure planning, which would benefit from a leaner and more data driven approach. I would welcome if the authors could include a reflection on the utility of framings in urban health decision making. Particular attention should be given to the ecological fallacy as populations are studied but individuals may participate in many populations and thus cumulate exposures or compensate them. This seems particular tricky if geographical approaches are used that do not include behavioural parameters.

 Many statements throughout the text and particularly section 2.4 seem to indicate that the authors acknowledge that cities are complex adaptive systems. This would imply that linear regression models and alike are tools that struggle to provide adequate information for decision making. This brings me to my main point of critique of the text in which I think, the field of medical epidemiology is well explored but expertise from ecology including its methods for analysis and modelling of complex ecosystems is largely missing. Secondly, in view of the polycrisis I would find it appropriate that urban health be concerned with other species than humans too, i.e. domestic animals, urban wildlife and plants, which in co-evolution and co-habitation with humans play a role in physical and mental human health but would also deserve some attention for their own sake. Understanding humans as part of nature and thus cities as dense ecosystems may shed some new understanding on public health issues, where species can co-benefit or co-suffer from exposures.

 A further point that may be explored is that surveillance should be retrofitted from the decision making process to provide the data that is needed to make informed decisions. Traditionally, we use available data to produce information. However, when complex systems are addressed, it becomes more important to identify and collect the data that is needed to observe and monitor health over time. Particularly, when health is co-constructed with citizens, it will require more and more varied data than the one we are used to collect in medical and health communities, which still rely largely on a deficit-oriented definition of health, i.e. absence of disease.

Is the review written in accessible language?

Yes

Are all factual statements correct and adequately supported by citations?

Yes

Are the conclusions drawn appropriate in the context of the current research literature?

Partly

Is the topic of the review discussed comprehensively in the context of the current literature?

Partly

Reviewer Expertise:

Veterinary Epidemiology, Participatory Modelling, One Health, Knowledge Integration

I confirm that I have read this submission and believe that I have an appropriate level of expertise to confirm that it is of an acceptable scientific standard, however I have significant reservations, as outlined above.

## References

[1] : Health beyond borders: the future of health promotion. *Scand J Public Health* .2024; 10.1177/14034948241288272 14034948241288272 10.1177/14034948241288272 39448891 PMC11951354

[2] : A health promotion perspective on One Health. *Can J Public Health* .2024;115(2) : 10.17269/s41997-024-00872-y 271-275 10.17269/s41997-024-00872-y 38478217 PMC11006651

# Response

**Published:** 2025-02-28

**Authors:** Michael Garber

**Affiliations:** University of California San Diego, California, USA

**Reviewer 3 (Simon Rüegg)**

 Reviewers comments

**Given that in the 21 ^st^ century most of the global population is living in urban settings, the reflection on the importance and requirements of urban epidemiology are pertinent and timely. Particularly the geographic resolution is a hindrance in open sight which must be brought to the attention of policy makers and health agents. However, given the current polycrisis including climate change, biodiversity loss and pollution which are directly and indirectly affecting human health, the sole emphasis on human health indicators seems somewhat anachronistic and narrowly focused. The authors legitimately build on historic practices in epidemiology using the four framings of cities as contexts, as determinants, as causes of human health and as complex systems that shape health.**

**I would find it interesting to differentiate somewhat more explicitly, which framing is useful for which kind of questions. Admittedly I am biased by a complexity approach to health. But I would advocate such an approach primarily for wicked health problems for which multiple aspects interact. Also, in the context of sustainable development participatory and complexity-aware approaches have shown to be more effective than linear analysis and intervention. There may however be public health related questions, e.g. health care infrastructure planning, which would benefit from a leaner and more data driven approach. I would welcome if the authors could include a reflection on the utility of framings in urban health decision making. Particular attention should be given to the ecological fallacy as populations are studied but individuals may participate in many populations and thus cumulate exposures or compensate them. This seems particular tricky if geographical approaches are used that do not include behavioural parameters.**

**Many statements throughout the text and particularly section 2.4 seem to indicate that the authors acknowledge that cities are complex adaptive systems. This would imply that linear regression models and alike are tools that struggle to provide adequate information for decision making. This brings me to my main point of critique of the text in which I think, the field of medical epidemiology is well explored but expertise from ecology including its methods for analysis and modelling of complex ecosystems is largely missing. Secondly, in view of the polycrisis I would find it appropriate that urban health be concerned with other species than humans too, i.e. domestic animals, urban wildlife and plants, which in co-evolution and co-habitation with humans play a role in physical and mental human health but would also deserve some attention for their own sake. Understanding humans as part of nature and thus cities as dense ecosystems may shed some new understanding on public health issues, where species can co-benefit or co-suffer from exposures.**

**A further point that may be explored is that surveillance should be retrofitted from the decision making process to provide the data that is needed to make informed decisions. Traditionally, we use available data to produce information. However, when complex systems are addressed, it becomes more important to identify and collect the data that is needed to observe and monitor health over time. Particularly, when health is co-constructed with citizens, it will require more and more varied data than the one we are used to collect in medical and health communities, which still rely largely on a deficit-oriented definition of health, i.e. absence of disease.**

 References

 1. de Leeuw E: Health beyond borders: the future of health promotion. *Scand J Public Health*. 2024. 14034948241288272 PubMed Abstract | Publisher Full Text

 2. de Leeuw E, Kickbusch I, Rüegg SR: A health promotion perspective on One Health. *Can J Public Health*. 2024; **115**(2): 271-275 PubMed Abstract | Publisher Full Text

**Authors Response: **We appreciate the reviewer's insightful comments and agree that a more integrated approach to urban health, encompassing human, animal, and environmental health, is crucial in the 21st century. We acknowledge that our current focus is primarily on human health, reflecting the traditional scope of epidemiology. Like Dr. Rüegg, we are interested in expanding this scope to better reflect the interconnectedness of human, animal, and environmental health, in line with the concepts of planetary health and One Health.

 To address Dr. Rüegg's concerns, we have made the following additions to the manuscript:

  

1. **Explicitly differentiate the utility of each framing (cities as contexts, determinants, causes, and complex systems) for different types of urban health questions.** This will clarify the specific applications of each perspective and guide researchers and policymakers in selecting the most appropriate approach for their needs.

**Added text**: We added the following text to Section 2.1:

 Measuring the magnitude of urban-health challenges through each of these lenses has value for researchers, policymakers, and advocates. For instance, viewing urban areas as *contexts* for health allows us to describe and compare health outcomes across different urban environments, highlighting disparities and identifying areas to prioritize for intervention. Understanding urban areas as *determinants*and *modifiers* of health enables us to investigate the causal impacts of specific urban characteristics and interventions on health outcomes and to inform action to change those characteristics. Finally, adopting a *complex systems* perspective allows us to appreciate the interconnectedness of factors influencing urban health, informing the development of holistic interventions at the structural level that can be mutually beneficial for many domains of population health, ecosystems, and climate resilience. ^1^

1. **Expand the discussion on complex systems to include the interconnectedness of human, animal, and environmental health.** This will acknowledge the broader ecological context of urban health and highlight the importance of considering these interrelationships when developing interventions and policies.

**Added text**: We added the following text at the end of the first paragraph of Section 2.4:

 In the context of urban health, a complex systems approach recognizes the intricate interplay between human health, animal health, and the health of the environment. This perspective acknowledges that human health is not isolated but rather embedded within a broader ecological context. Specific conceptual frameworks that make this interconnectedness explicit include the One Health and planetary health. ^1,2^ For example, seen through a planetary-health prism, urban green spaces not only provide recreational opportunities and promote physical activity, but also contribute to biodiversity, air quality, and climate regulation, benefiting both human, animal health, and mitigation of adaptation to climate change.

1. **Include a brief discussion of the ecological fallacy and its potential implications for urban health research.** This will emphasize the need for careful interpretation of group-level data and caution against making inferences about individuals based on aggregate-level observations.

**Added text**: We added the following text to Section 2.3 when discussing limitations and cautions of quasi-experimental methods:

 Fifth, quasi-experimental methods do not necessarily alleviate long-recognized concerns regarding the interpretation of group-level associations to individuals. ^3^ The ecological fallacy can occur when associations observed at the group level are assumed to hold at the individual level. More technically, it can occur when the joint distribution of the exposure and outcome is not available at the individual level, and group-level summaries are used as proxies for this individual-level distribution. The target level of inference—individuals vs population distributions—remains important when using these quasi-experimental methods.

1. **Acknowledge the limitations of linear regression models for analyzing complex systems and suggest alternative modeling approaches.** This will encourage the exploration of more sophisticated methods that can better capture the dynamic interactions and feedback loops inherent in urban health systems.

**Added text:** We added the following text at the beginning of the third paragraph of Section 2.4:

 While traditional statistical methods, such as linear regression, can be useful for analyzing certain aspects of urban health, they may be limited for capturing the complexity of these systems. Alternative modeling approaches, such as system dynamics models or agent-based models (ABMs), may be more suitable for simulating and understanding the dynamic behavior of urban health systems.

**Summary notes:**

 With these additions, we believe our manuscript is strengthened and better reflects the complex and interconnected nature of urban health in the 21st century.

**References cited in this response document**

 Kindly note that the reference order differs from that of the article.

 1.             Whitmee S, Haines A, Beyrer C, et al. Safeguarding human health in the Anthropocene epoch: report of The Rockefeller Foundation–Lancet Commission on planetary health. *The Lancet*. 2015;386(10007):1973-2028. doi:10.1016/S0140-6736(15)60901-1

 2.             De Castañeda RR, Villers J, Guzmán CAF, et al. One Health and planetary health research: leveraging differences to grow together. *The Lancet Planetary Health*. 2023;7(2):e109-e111. doi:10.1016/S2542-5196(23)00002-5

 3.             Wakefield J. Ecologic Studies Revisited. *Annu Rev Public Health*. 2008;29(1):75-90. doi:10.1146/annurev.publhealth.29.020907.090821

# Reviewer report: Reviewer response for version 1

**DOI:** 10.5256/f1000research.170075.r337108

**Published:** 2024-11-21

**Authors:** Gregory Heath

**Affiliations:** 1 Department of Health and Human Performance, Graduate Program in Public Health, The University of Tennessee at Chattanooga and the University of Tennessee Health Science Center College of Medicine Chattanooga, Chattanooga, Tennessee, USA

The authors provide a good overview of the position that urban health is critical to global health particularly relative to emerging economies among low to middle income countries, where there is a significant shift in populations moving from rural to urban centers. 

 This is compounded by the existence of what is referred to as the epidemiologic transition from primarily infectious disease to non-communicable diseases (NCD) such as cardiovascular disease, T2DM, HTN, and their associated risk factors of obesity, physical inactivity, and unhealthy refined dietaries. 

 The authors appropriately identify the importance of descriptive epidemiology and public health surveillance to understand these population dynamics.  Their emphasis on using such surveillance to track not only diseases and their biological risk factors but also policies and practices supporting environmental supports for healthy living within the context of the urban environment are key to protecting and promoting the health of urban populations.  The paucity of such policy and practice surveillance is mentioned as a current deficiency within urban environments.  Hence, the authors provide insights and a sense of urgency for the greater adoption of systems thinking in tackling urban population health, where intersectoral collaboration, coordination, and cooperation are currently often lacking both in intention and function. 

 The authors should cite additional sources of local health and policy data from the following sources:

 The CDC Places data - that provides small area epidemiologic surveillance across sectors:

  https://www.cdc.gov/places/index.html 

 Also, the County Health Rankings published by the University of Wisconsin's Population Health Institute should also be cited:  https://www.countyhealthrankings.org/

Is the review written in accessible language?

Yes

Are all factual statements correct and adequately supported by citations?

Yes

Are the conclusions drawn appropriate in the context of the current research literature?

Yes

Is the topic of the review discussed comprehensively in the context of the current literature?

Yes

Reviewer Expertise:

Guerry Professor Emeritus in the Public Health and Medicine at the University of Tennessee Chattanooga and a former Lead Health Scientist/epidemiologist at the U.S. Centers for Disease Control and Prevention (CDC), for over 20 years.  A doctorate in physiology/nutrition and an MPH in epidemiology from the Loma Linda University School of Public Health.  I have spent most of my career devoted to the epidemiology, prevention, and management of chronic diseases and their associated behavioral risk factors (e.g., physical inactivity, poor diet, obesity).  I am widely published in the preventive medicine and public health literature with an h-index of 65.  I am a Fellow in the American College of Sports Medicine (ACSM) and the American Heart Association’s Council on Epidemiology and Prevention.

I confirm that I have read this submission and believe that I have an appropriate level of expertise to confirm that it is of an acceptable scientific standard, however I have significant reservations, as outlined above.

## References

[1] : Creating healthy and sustainable cities: what gets measured, gets done. *Lancet Glob Health* .2022;10(6) : 10.1016/S2214-109X(22)00070-5 e782-e785 10.1016/S2214-109X(22)00070-5 35561709

[2] : Achieving the SDGs: Evaluating indicators to be used to benchmark and monitor progress towards creating healthy and sustainable cities. *Health Policy* .2020;124(6) : 10.1016/j.healthpol.2019.03.001 581-590 10.1016/j.healthpol.2019.03.001 30935701

[3] : Urban health: an example of a "health in all policies" approach in the context of SDGs implementation. *Global Health* .2019;15(1) : 10.1186/s12992-019-0529-z 87 10.1186/s12992-019-0529-z 31856877 PMC6924052

[4] : Parks, Trails, and Greenways for Physical Activity: A Community Guide Systematic Economic Review. *Am J Prev Med* .2024;66(6) : 10.1016/j.amepre.2024.01.021 1089-1099 10.1016/j.amepre.2024.01.021 38331114 PMC11102847

[5] : Environmental, health, wellbeing, social and equity effects of urban green space interventions: A meta-narrative evidence synthesis. *Environ Int* .2019;130: 10.1016/j.envint.2019.104923 104923 10.1016/j.envint.2019.104923 31228780

[6] : Intersectoral Policy Priorities for Health.2017;30212153

# Response

**Published:** 2025-02-28

**Authors:** Michael Garber

**Affiliations:** University of California San Diego, California, USA

Reviewer 2 (Gregory Heath)

**R2. Overview. **The authors provide a good overview of the position that urban health is critical to global health particularly relative to emerging economies among low to middle income countries, where there is a significant shift in populations moving from rural to urban centers. 

 This is compounded by the existence of what is referred to as the epidemiologic transition from primarily infectious disease to non-communicable diseases (NCD) such as cardiovascular disease, T2DM, HTN, and their associated risk factors of obesity, physical inactivity, and unhealthy refined dietaries.

 The authors appropriately identify the importance of descriptive epidemiology and public health surveillance to understand these population dynamics.  Their emphasis on using such surveillance to track not only diseases and their biological risk factors but also policies and practices supporting environmental supports for healthy living within the context of the urban environment are key to protecting and promoting the health of urban populations.  The paucity of such policy and practice surveillance is mentioned as a current deficiency within urban environments.  Hence, the authors provide insights and a sense of urgency for the greater adoption of systems thinking in tackling urban population health, where intersectoral collaboration, coordination, and cooperation are currently often lacking both in intention and function. 

**R2. Overview. Response: **We appreciate Dr. Heath’s thoughtful summary of our paper and his suggestions to include the additional citations for sources of local health and policy data.

**R2.1.** The authors should cite additional sources of local health and policy data from the following sources:

 The CDC Places data - that provides small area epidemiologic surveillance across sectors:

  https://www.cdc.gov/places/index.html 

 Also, the County Health Rankings published by the University of Wisconsin's Population Health Institute should also be cited: https://www.countyhealthrankings.org/

**R2.1. Response: **We appreciate the suggestion to add these resources. We’ve added a reference to CDC PLACES in the section on small-area estimation:

**Quote from text: **CDC PLACES is another large-scale application of SAE methods in the U.S. context, estimating various chronic-disease-related measures at the sub-county level. ^1^

**R2.1. Response, continued: **And we’ve added a reference to the U Wisconsin resource in the “Actions needed” section, by revising the following sentence:

**Quote from text:** U.S.-based examples of such tools include New York University’s City Health Dashboard. ^2,3^ and the University of Wisconsin’s County Health Rankings. ^4^

 References

 1. Giles-Corti B, Moudon AV, Lowe M, Adlakha D, et al.: Creating healthy and sustainable cities: what gets measured, gets done. *Lancet Glob Health*. 2022; **10**(6): e782-e785 PubMed Abstract | Publisher Full Text

 2. Giles-Corti B, Lowe M, Arundel J: Achieving the SDGs: Evaluating indicators to be used to benchmark and monitor progress towards creating healthy and sustainable cities. *Health Policy*. 2020; **124** (6): 581-590 PubMed Abstract | Publisher Full Text

 3. Ramirez-Rubio O, Daher C, Fanjul G, Gascon M, et al.: Urban health: an example of a "health in all policies" approach in the context of SDGs implementation. *Global Health*. 2019; **15**(1): 87 PubMed Abstract | Publisher Full Text

 4. Jacob V, Reynolds JA, Chattopadhyay SK, Hopkins DP, et al.: Parks, Trails, and Greenways for Physical Activity: A Community Guide Systematic Economic Review. *Am J Prev Med*. 2024; **66** (6): 1089-1099 PubMed Abstract | Publisher Full Text

 5. Hunter RF, Cleland C, Cleary A, Droomers M, et al.: Environmental, health, wellbeing, social and equity effects of urban green space interventions: A meta-narrative evidence synthesis. *Environ Int*. 2019; **130**: 104923 PubMed Abstract | Publisher Full Text

 6. Watkins DA: Intersectoral Policy Priorities for Health. 2017.

**References cited in this response document**

 Kindly note that the reference order differs from that of the article.

 1.             Greenlund KJ, Lu H, Wang Y, et al. PLACES: Local Data for Better Health. *Prev Chronic Dis*. 2022;19:210459. doi:10.5888/pcd19.210459

 2.             City Health DASHBOARD. Accessed May 13, 2024. https://www.cityhealthdashboard.com

 3.             Thorpe LE, Gourevitch MN. Data Dashboards for Advancing Health and Equity: Proving Their Promise? *Am J Public Health*. 2022;112(6):889-892. doi:10.2105/AJPH.2022.306847

 4. *2024 National Findings Report  Creating Thriving Communities Through Civic Participation*. University of Wisconsin Public Health Institute School of Medicine and Public Health; 2024. Accessed January 30, 2025. https://www.countyhealthrankings.org/findings-and-insights/2024-national-findings-report

# Reviewer report: Reviewer response for version 1

**DOI:** 10.5256/f1000research.170075.r324311

**Published:** 2024-10-08

**Authors:** Alina Schnake-Mahl

**Affiliations:** 1 Urban Health Collaborative, Department of Health Management and Policy, Drexel Dornsife School of Public Health, Philadelphia, USA

This article provides a structured framework for thinking about epidemiology and urban health, and opportunities for future epidemiologic work in this area. I commend authors for taking on the difficult but important task of outlining the epi case for urban health. The article is well organized, comprehensive, and provides a nice overview of current work. My suggestions are mostly small additions.

 Cities and urban are used interchangeably- while other work such as the urban health textbook, have I believe worked to define these terms, it would be helpful to repeat or summarize the definition of urban and city for readers.  

 The sentence “ Variable exposure to environmental conditions (both social and physical) gives rise to health inequities in urban populations by characteristics such as income and wealth, residential neighborhood, race and ethnicity, age, disability, gender identity, sexual orientation, or migration status. ^16^ “ seems to orient around individual level predictors of health inequities, rather than inequitable social, political, and environmental exposures as predictors of inequities. Both should probably be included.

 Though the section on small area estimates is within the descriptive epidemiology section, it’s important to add limitations of small area estimation for sub-unit evaluation of health changes over time, as the methods integrate sub-unit specific information and therefor shouldn’t be used for assessing changes or evaluating local intervention effects over time, and any use with modeling should incorporate the uncertainty estimates. See, for example the language on the CDC PLACES website about limitations of using the data.  It may be worth mentioning potential of Bayesian methods for small areas as well?

 The section on tracking policy would benefit from acknowledging and briefly describing legal epidemiology and policy surveillance methods as burgeoning and rigorous methods to track laws and policies over time. However, local policies are often difficult to track especially historically, despite resources such as Municode, in the United States, because local laws and policies are often unavailable on city or county websites or tracking sources. This is also a good opportunity to highlight the importance of interdisciplinary collaborations (e.g. with lawyers or policy experts) to identify, code, and interpret laws and policies, while bringing our epidemiologic focus on sources of bias, good coding and data management practices, and fascination with methodological rigor.  

-
  - See for example (refer 1 and refer 2)

The paragraph on tracking policies would also benefit from citations focused on tracking city-level (or sub city-level) policies. Most of the citations are focused on state level (or multilevel – e.g. the matthay article) articles. Suggested citation:

- refer 3

It would be helpful to allude back to the issue of lack of outcome data at the city or sub-city level for quasi-experimental evaluation. It would also be helpful to mention the importance of understanding  effect modification within a city (e.g. by neighborhood SES or SVI) to monitor impacts of city level policies on disparities.

Is the review written in accessible language?

Yes

Are all factual statements correct and adequately supported by citations?

Yes

Are the conclusions drawn appropriate in the context of the current research literature?

Yes

Is the topic of the review discussed comprehensively in the context of the current literature?

Yes

Reviewer Expertise:

social epidemiology, policy evaluation, urban health, health disparities

I confirm that I have read this submission and believe that I have an appropriate level of expertise to confirm that it is of an acceptable scientific standard, however I have significant reservations, as outlined above.

## References

[1] : Law and urban governance for health in times of rapid change. *Health Promot Int* .2021;36(Supplement_1) : 10.1093/heapro/daab064 i4-i12 10.1093/heapro/daab064 34897442 PMC8667544

[2] : Policy Surveillance: A Vital Public Health Practice Comes of Age. *J Health Polit Policy Law* .2016;41(6) : 10.1215/03616878-3665931 1151-1173 10.1215/03616878-3665931 27531941

[3] : Beyond minimum wage: Broader employment policies can significantly affect food insecurity. *Translational Behavioral Medicine* .2024;14(6) : 10.1093/tbm/ibae022 333-337 10.1093/tbm/ibae022 PMC1210414838734881

# Response

**Published:** 2025-02-28

**Authors:** Michael Garber

**Affiliations:** University of California San Diego, California, USA

Reviewer 1 (Alina Schnake-Mahl)

**R1. Overview. **This article provides a structured framework for thinking about epidemiology and urban health, and opportunities for future epidemiologic work in this area. I commend authors for taking on the difficult but important task of outlining the epi case for urban health. The article is well organized, comprehensive, and provides a nice overview of current work. My suggestions are mostly small additions.

**R1. Overview. Response: **We thank Dr. Schnake-Mahl for her kind comments and for her helpful feedback on our article. We made several changes in response to her feedback, as detailed point by point below. We believe our article has improved as a result of these changes.

**R1.1.** Cities and urban are used interchangeably- while other work such as the urban health textbook, have I believe worked to define these terms, it would be helpful to repeat or summarize the definition of urban and city for readers.

**R1.1. Response: **We agree that it would be helpful to discuss these terms more explicitly. We added a few sentences on this in the introduction:

**Quote from revised text: **As a preliminary note on language, an *urban area* has been defined “as a geographical space characterized by a continuous urban settlement.” ^1^ The phrase is commonly used in a more general sense than *city*, which can refer to a discrete urban area defined with administrative boundaries. ^2^ For a rigorous discussion of these terms, we recommend Chapter 1 of Urban Public Health by Diez-Roux ^2^ and Tonne et al (2021). ^3^ In this article, we usually use the more general term— *urban area*—but occasionally use the word *city* as a rhetorical shorthand.

**R1.2.** The sentence “ Variable exposure to environmental conditions (both social and physical) gives rise to health inequities in urban populations by characteristics such as income and wealth, residential neighborhood, race and ethnicity, age, disability, gender identity, sexual orientation, or migration status. ^16^ “ seems to orient around individual level predictors of health inequities, rather than inequitable social, political, and environmental exposures as predictors of inequities. Both should probably be included.

**R1.2. Response: **We appreciate Dr. Schnake-Mahl raising that this sentence evoked this implication. It was not our intention to focus on individual-level characteristics as causes of inequities. We have slightly revised this sentence to more clearly assert that the causes of inequities tend to be structural (above-the-individual) factors:

**Quote from revised text: “**Unequal exposure over time to social, political, and environmental conditions gives rise to health inequities in urban populations by characteristics such as income and wealth **…”**

**R1.3.** Though the section on small area estimates is within the descriptive epidemiology section, it’s important to add limitations of small area estimation for sub-unit evaluation of health changes over time, as the methods integrate sub-unit specific information and therefor shouldn’t be used for assessing changes or evaluating local intervention effects over time, and any use with modeling should incorporate the uncertainty estimates. See, for example the language on the CDC PLACES website about limitations of using the data.  It may be worth mentioning potential of Bayesian methods for small areas as well?

**R1.3. Response**: We thank Dr. Schnake-Mahl for suggesting that we add the caution of using SAE methods for assessing changes over time. Upon consulting CDC PLACES materials, as suggested, we’ve added the following sentence:

**Quote from revised text: **Finally, caution is recommended when using small-area estimation to monitor changes over time, ^4^ as the methods may involve inputs with a coarse temporal resolution and because the model inputs may assume time-invariant predictor-outcome associations.

**R1.3. Response, continued: **We also agree with the suggestion to mention Bayesian methods. We’ve added a citation to an article that summarizes development in SAE and disease mapping through a Bayesian lens (bold denotes addition).

**Quote from revised text with addition underlined: **SAE methods include, for example, non-parametric methods that use measured associations between the outcome and demographic characteristics at the larger geographic unit (e.g., U.S. states) and apply those associations to smaller units where the outcome is not measured (such as counties), spatial data smoothing, ^5^ regression techniques using area-specific data as predictors, ^6^ **and an array of Bayesian techniques**. ^7^

**R1.4. **The section on tracking policy would benefit from acknowledging and briefly describing legal epidemiology and policy surveillance methods as burgeoning and rigorous methods to track laws and policies over time. However, local policies are often difficult to track especially historically, despite resources such as Municode, in the United States, because local laws and policies are often unavailable on city or county websites or tracking sources. This is also a good opportunity to highlight the importance of interdisciplinary collaborations (e.g. with lawyers or policy experts) to identify, code, and interpret laws and policies, while bringing our epidemiologic focus on sources of bias, good coding and data management practices, and fascination with methodological rigor.  

-
  - See for example (refer 1 and refer 2)

**R1.4. Response: **We again thank Dr. Schnake-Mahl for the helpful suggestion, as we had limited familiarity with legal epidemiology and the resources she suggested. We read these suggested references and made major revisions to this part of the text. The paragraph beginning with “Related to the need for…” in Section 2.2.2 has been rewritten almost entirely guided by these suggestions.

**R1.5. **The paragraph on tracking policies would also benefit from citations focused on tracking city-level (or sub city-level) policies. Most of the citations are focused on state level (or multilevel – e.g. the matthay article) articles. Suggested citation:

- refer 3

**R1.5. Response: **Again, we appreciate Dr. Schnake-Mahl’s attention to this detail. In this section, we’ve omitted our citation to the Matthay article in this particular section—although we still cite it in discussing challenges of co-occurring policies. And we now include more examples of city-specific policy surveillance.

**R1.6. **It would be helpful to allude back to the issue of lack of outcome data at the city or sub-city level for quasi-experimental evaluation. It would also be helpful to mention the importance of understanding effect modification within a city (e.g. by neighborhood SES or SVI) to monitor impacts of city level policies on disparities.

**R1.6. Response:** On the first point, we agree. We’ve added the following sentence to Section 2.3 when discussing challenges with quasi-experimental methods

**Quote from text:** Fourth, as emphasized in Section 2.2., longitudinally measured data on both the intervention and outcome are often not available, underscoring the need for sound surveillance.

**R1.6. Response: **Regarding the second point (effect modification), we had noted the opportunity for assessment of effect modification, but we agree that this could have been emphasized more. We’ve added another sentence on the importance of effect modification

**Quote from text (bold denotes addition):** Some of these tools allow for the estimation of place-specific effects which can inform the extent to which intervention effects are modified **by social or environmental context**. ^8^ **Understanding within-city effect modification can help to monitor impacts of city-level actions on disparities.**

 References

 1. Burris S, Lin V: Law and urban governance for health in times of rapid change. *Health Promot Int*. 2021; **36**(Supplement_1): i4-i12 PubMed Abstract| Publisher Full Text

 2. Burris S, Hitchcock L, Ibrahim J, Penn M, et al.: Policy Surveillance: A Vital Public Health Practice Comes of Age. *J Health Polit Policy Law*. 2016; **41** (6): 1151-1173 PubMed Abstract | Publisher Full Text

 3. Piekarz-Porter E, Oddo V, Stiehl E, Kim S: Beyond minimum wage: Broader employment policies can significantly affect food insecurity. *Translational Behavioral Medicine*. 2024; **14** (6): 333-337 Publisher Full Text

**References cited in this response document**

 Kindly note the reference order differs from that of the article.

 1.             Mela A. Urban Areas. In: *Encyclopedia of Quality of Life and Well-Being Research*. Springer, Dordrecht; 2014:6826-6828. doi:10.1007/978-94-007-0753-5_3122

 2.             Diez-Roux AV. What Is Urban Health? Defining the Geographic and Substantive Scope. In: Lovasi GS, Diez Roux AV, Kolker J, eds. *Urban Public Health: A Research Toolkit for Practice and Impact*. Oxford University Press; 2020:3-26. doi:10.1093/oso/9780190885304.003.0001

 3.             Tonne C, Adair L, Adlakha D, et al. Defining pathways to healthy sustainable urban development. *Environment International*. 2021;146:106236. doi:10.1016/j.envint.2020.106236

 4.             Greenlund KJ, Lu H, Wang Y, et al. PLACES: Local Data for Better Health. *Prev Chronic Dis*. 2022;19:210459. doi:10.5888/pcd19.210459

 5.             Mercer L, Wakefield J, Chen C, Lumley T. A comparison of spatial smoothing methods for small area estimation with sampling weights. *Spatial Statistics*. 2014;8:69-85. doi:10.1016/j.spasta.2013.12.001

 6.             Jia H, Muennig P, Borawski E. Comparison of small-area analysis techniques for estimating county-level outcomes. *American Journal of Preventive Medicine*. 2004;26(5):453-460. doi:10.1016/j.amepre.2004.02.004https://f1000research.com/articles/13-950/v1#

 7.             MacNab YC. Bayesian disease mapping: Past, present, and future. *Spatial Statistics*. 2022;50:100593. doi:10.1016/j.spasta.2022.100593

 8.             Garber MD. Precision and Weighting of Effects Estimated by the Generalized Synthetic Control and Related Methods: The Case of Medicaid Expansion. *Epidemiology*. 2024;35(2):273-277. doi:10.1097/EDE.0000000000001702
